# Protecting the Eye Lens from Oxidative Stress through Oxygen Regulation

**DOI:** 10.3390/antiox12091783

**Published:** 2023-09-20

**Authors:** Witold Karol Subczynski, Marta Pasenkiewicz-Gierula, Justyna Widomska

**Affiliations:** 1 Department of Biophysics, Medical College on Wisconsin, Milwaukee, WI 53226, USA; 2 Department of Computational Biophysics and Bioinformatics, Jagiellonian University, 30-387 Krakow, Poland; marta.pasenkiewicz-gierula@uj.edu.pl; 3 Department of Biophysics, Medical University of Lublin, 20-090 Lublin, Poland

**Keywords:** eye lens, oxygen partial pressure, oxygen concentration, lens lipids, barriers to oxygen transport, oxygen consumption

## Abstract

Molecular oxygen is a primary oxidant that is involved in the formation of active oxygen species and in the oxidation of lipids and proteins. Thus, controlling oxygen partial pressure (concentration) in the human organism, tissues, and organs can be the first step in protecting them against oxidative stress. However, it is not an easy task because oxygen is necessary for ATP synthesis by mitochondria and in many biochemical reactions taking place in all cells in the human body. Moreover, the blood circulatory system delivers oxygen to all parts of the body. The eye lens seems to be the only organ that is protected from the oxidative stress through the regulation of oxygen partial pressure. The basic mechanism that developed during evolution to protect the eye lens against oxidative damage is based on the maintenance of a very low concentration of oxygen within the lens. This antioxidant mechanism is supported by the resistance of both the lipid components of the lens membrane and cytosolic proteins to oxidation. Any disturbance, continuous or acute, in the working of this mechanism increases the oxygen concentration, in effect causing cataract development. Here, we describe the biophysical basis of the mechanism and its correlation with lens transparency.

## 1. Introduction

To perform their function of focusing pictures of surrounding objects on the retina, the eye lens and cornea must be transparent throughout the entire human life. Avascularity of the lens and the cornea is one of the ways to maintain the transparency. Another is diminishing the scattering of incoming light. For that purpose, the fiber cells comprising the lens lose their cytoplasmic organelles during maturation [1,2,3]. Only the most superficial and not yet matured layers of cortical fiber cells still contain organelles, including mitochondria [1,4]. Because the lens is avascular, the metabolites needed for biochemical reactions in the central part of the lens have to be delivered from the surface by diffusion; the fiber cell metabolism is thus diminished to the very minimum. One of the metabolites is the primary oxidant, molecular oxygen. In contrast with the lens, the cornea obtains enough oxygen for its metabolism directly from the air [5].

This review is focused on three mechanisms developed during evolution that control oxygen partial pressure within the lens and ensure that it remains at a very low level throughout the entire human life. They achieve their goals by (1) controlling the oxygen partial pressure outside the lens, (2) consuming oxygen within the lens, and (3) utilizing and modifying the barriers to oxygen transport into the lens center. These three direct antioxidant direct mechanisms are assisted by the resistance of the lipid components of the eye lens membrane and cytosolic proteins to oxidation.

## 2. Oxygen Partial Pressure and Oxygen Concentration

**Figure 1:**
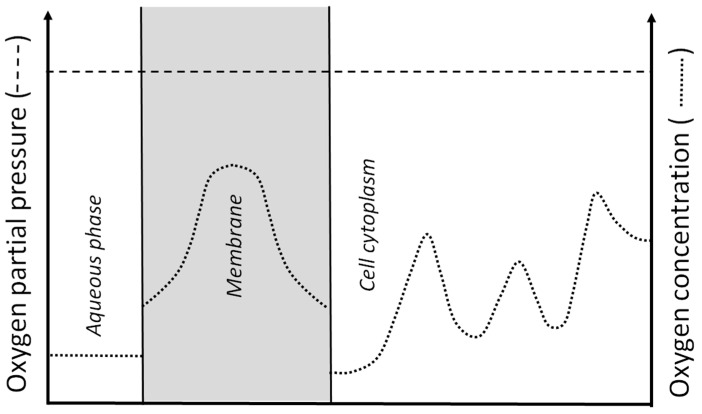
Schematic illustration of the local oxygen partial pressure (--------) and the local oxygen concentration (········) across the sample (aqueous environment, cell membrane, and cell cytoplasm). The sample is in equilibrium with the set oxygen partial pressure, which is the same across the sample, while the local oxygen concentration depends on the local solubility of the oxygen in the environment (as indicated in the drawing). It should be noted that the driving force for the oxygen flux is not the difference in oxygen concentration; rather, it is the difference in the oxygen partial pressure. This figure illustrates the scenario in which the system is in equilibrium, and the partial pressure of the oxygen is the same at all points in the system, but the local oxygen concentrations differ significantly. In this system, the net oxygen transport between the different regions does not occur.

Generally, the content of oxygen in the investigated systems is described either by the oxygen concentration or the oxygen partial pressure [6]. The quantities are related to and can be derived from each other. The oxygen partial pressure in the system equals the oxygen pressure in the gaseous phase with which the system should be equilibrated to obtain a measured value. Two units are most commonly used in medicine: mmHg and percentage of oxygen. The partial pressure of oxygen in the air at 1 atmosphere is 156 mmHg or 20.9%. In research on the eye, mmHg is used as a unit of the partial pressure. For systems in equilibrium, the oxygen partial pressure is the same at any point in the system. This part of the system can be the aqueous phase outside the cell, the cell membrane, or the cytoplasm. In contrast, the oxygen concentration (mols per liter) for systems in equilibrium is determined by the oxygen partial pressure and the oxygen solubility coefficient at any spot in the system. Thus, the oxygen partial pressure across a system in equilibrium is the same, whereas the oxygen concentration can differ significantly. The oxygen partial pressure determines not only the local oxygen concentration but also the direction of the oxygen flux (from greater to lower oxygen partial pressure). This is not true for the oxygen concentration (see Figure 1 for further explanation).

It is rational to assume that it is easier to remove oxygen from a system with a lower oxygen concentration (i.e., with a low oxygen solubility coefficient) than otherwise. In biological objects, oxygen can be removed by an oxygen consumption mechanism. Removing oxygen from the aqueous phase requires lower oxygen consumption rates than from a dense membrane system. We believe that a clear understanding of these concepts is significant for the explanation of the oxygen distribution around and within the eye lens. 

## 3. Oxygen Partial Pressure Outside the Lens (First Mechanism)

**Figure 2:**
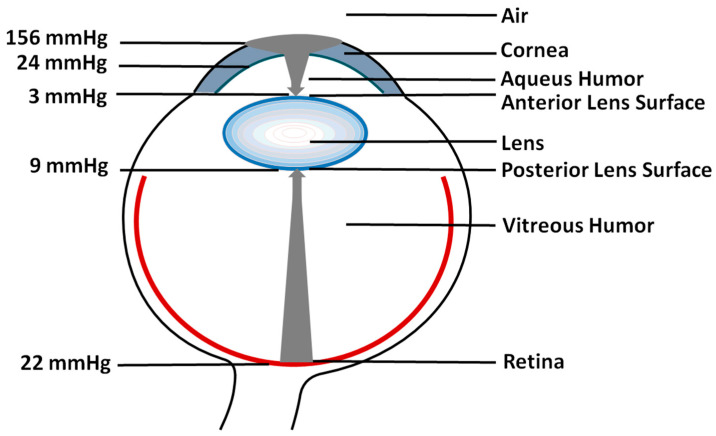
Schematic drawing showing the distribution of oxygen partial pressure in a healthy eye. Gray arrows show the oxygen flux from air to the anterior lens surface and from the retina to the posterior lens surface. Arrowheads indicate the changes in oxygen partial pressure toward the lens surface, with a thickness proportional to the partial pressure value (Note the change from 156 mmHg to 24 mmHg across the cornea, where the oxygen flux is consumed by mitochondria).

Maintaining low oxygen partial pressure at the lens surface is the first and most important step in protecting the eye lens from cataract development. In a healthy eye, the oxygen partial pressure at the lens surface is already very low. At the anterior surface (in the aqueous humor), the reported values are ~3 mmHg (Figure 2). On the opposite side of the lens, at the posterior surface (in the vitreous humor), the reported values are somewhat higher, of ~9 mmHg [7,8,9,10].

Oxygen diffuses from cornea through the aqueous humor to reach the anterior surface of the lens. The cornea is avascular and obtains oxygen directly from the air; the oxygen partial pressure at the cornea surface under the open eye conditions is 156 mmHg, and when the eye is closed, it drops to ~50 mmHg. So, the barrier for the transport of oxygen from the air to the anterior area must be very effective to enable the decrease in the oxygen partial pressure at the anterior surface to a value as low as 3 mmHg. The major barrier is within the cornea itself, where the oxygen partial pressure drops from 156 mmHg to 24 mmHg (Figure 2). Oxygen in the cornea is effectively consumed mainly by mitochondria at a rate of about 5 µL O_2_/mm^2^ cornea/hour [11,12]. A further drop in the oxygen partial pressure occurs mainly in the lens’ epithelial cell layer due to oxygen consumption by mitochondria [13,14,15].

On the other side of the lens, oxygen diffuses from the retina through the vitreous humor toward the posterior surface of the lens. The oxygen partial pressure in the vitreous humor, near the retina, is ~22 mmHg. It drops to a value of 9 mmHg at the posterior lens surface. This drop occurs during oxygen diffusion through the vitreous gel, mainly due to the ascorbate-dependent oxygen consumption reaction. The concentration of ascorbic acid in the intact vitreous is very high. Any disturbance in the oxygen partial pressure at the lens surface (acute or chronic), as reported after vitrectomy or hyperbaric oxygen treatment, results in the development of cataract. Thus, maintaining a low oxygen partial pressure outside the lens (i.e., at the lens surface) is the major mechanism to prevent cataract development.

For comparison, it is good practice to compare the values of the partial pressure of oxygen around the lens to the partial pressure of oxygen in typical tissue. The values of these partial pressures presented in the recent review [16] are 30–48 mmHg for brain tissue, 55.5 mmHg for liver tissue, ~72 mmHg for kidney tissue, and ~30 mmHg for muscle fibers. In hypoxic tumors, the partial pressure of oxygen can be as low as 9.6 mmHg in renal carcinomas [17], 6 mmHg in liver tumors [18], and 2.6 mmHg in primary brain tumors [18]. Normal oxygenation of brain tissue is assumed when the oxygen partial pressure reaches 35 mmHg [19].

## 4. Oxygen Consumption within the Lens (Second Mechanism)

**Figure 3:**
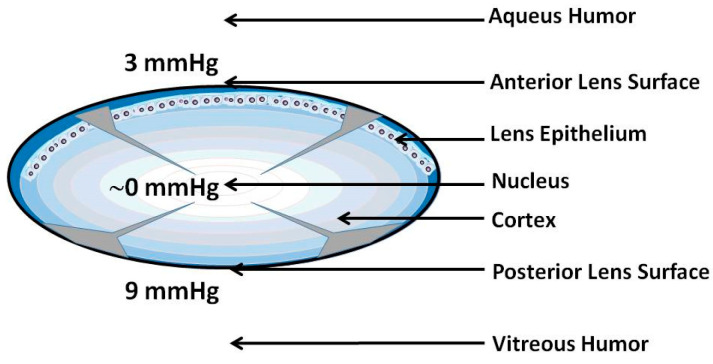
Schematic drawing showing the purported distribution of the oxygen partial pressure in a healthy eye lens. The values of the oxygen partial pressure at the surface of the anterior and posterior cortex of the lens in a healthy eye are taken from [7]. Arrowheads indicate the purported changes of the oxygen partial pressure toward the lens center, with the thickness proportional to the partial pressure value. McNulty et al. [10] reported that ~90% of oxygen flux from the lens surface to the lens center is consumed by the mitochondria located in the most superficial layers of not yet maturated fiber cells. The eye lens cortex (blue) and nucleus (white) are indicated. Differentiating fiber cells near the lens surface (dark blue) contain a normal complement of organelles, including mitochondria. Maturate fiber cells located deeper in the central region of the lens (light blue in cortex and white in nucleus) do not contain mitochondria.

Low oxygen partial pressure outside the lens does not guarantee that the partial pressure inside the eye lens is also very low (close to zero). To ensure that the partial pressure is low, oxygen must be consumed within the lens; otherwise, a steady flux of oxygen from the posterior surface (with an oxygen partial pressure of 9 mmHg) to the anterior surface (with an oxygen partial pressure of 3 mmHg) would be established [7,20]. It was shown that the outermost layers of the cortical fiber cells, i.e., those not yet maturated and containing mitochondria, can consume 90% of oxygen coming to the lens [10]. Thus, mitochondrial respiration contributes significantly to keeping the oxygen partial pressure low within the lens (Figure 3). Additionally, non-mitochondrial oxygen removal via ascorbate-dependent oxygen consumption [10,21] or glutathione-dependent oxygen consumption [8] in the lens nucleus helps to lower the oxygen partial pressure in this region to a level even below that in the cortex (Figure 3).

## 5. Barriers for Oxygen Transport into the Lens Center (Third Mechanism)

**Figure 4:**
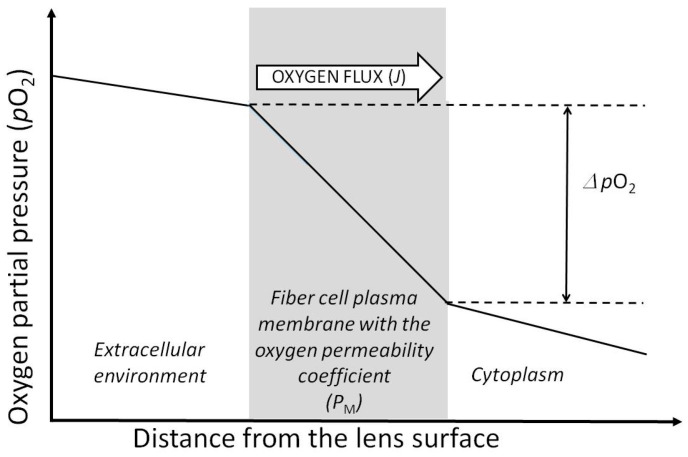
Oxygen partial pressure difference (∆*p*O_2_) across the fiber cell membrane with the oxygen permeability coefficient *P*_M_ formed by the oxygen consumption on one side of the membrane with a rate of *J*. The oxygen permeability coefficient across the membrane, *P*_M_, connects the oxygen flux, *J*, across the lipid bilayer with a difference in oxygen partial pressure on either side of the bilayer, ∆*p*: *J* = −*P*_M_∆*p*O_2_.

Another significant factor that helps maintain very low oxygen partial pressure within the lens is the set of barriers to oxygen diffusion from the lens surface to the lens center. On its way to the center, oxygen crosses thousands of fiber cell membranes. Each of the membranes is a small barrier. The barriers, together with the oxygen consumption (see Section 4 and Figure 3) contribute to the total oxygen partial pressure gradient across the eye lens (Figure 4). As the height of the barrier to oxygen membrane permeation is the inverse of the oxygen permeability coefficient across the membrane, *P*_M_, the oxygen partial pressure difference across the membrane is determined by the oxygen consumption rate on one side of the membrane and the oxygen permeability coefficient of the membrane (see Figure 4 for further explanation).

The oxygen permeability coefficient across the membrane depends on the membrane constituents. It was shown that fiber cell plasma membranes, with their high cholesterol content and high density of integral membrane proteins, constitute an effective permeability barrier [22,23,24,25,26] These barriers are higher in the lens nucleus than in the cortex. This is because the nucleus cells are older. With age, the lipid composition of the fiber cell membranes changes, the content of sphingolipids increases, and the content of phosphatidylcholine decreases [23,24,25,26]. This is accompanied by a pronounced increase in the cholesterol content [27,28,29,30]. The membranes of fiber cells are loaded with integral membrane proteins, and the load increases with age [31,32,33]. These proteins can form domains, arrays, and other structures [34,35,36,37], which, in turn, affect the organization of the membrane’s lipid bilayer component. Two major lipid domains induced by integral membrane proteins are boundary lipids and trapped lipids. The oxygen permeability coefficients of these domains are much lower than that of bulk lipids [38]. However, the high oversaturating amount of cholesterol in the bulk lipid domains leads to the formation of pure cholesterol bilayer domains whose oxygen permeability coefficient is low [38,39]. The coefficient is also low for cholesterol saturated bulk lipid domains [40]. Interestingly, the age-related changes in the membranes of the eye lens fiber cells are much greater than those in membranes of other organs and tissue.

A comparison of the oxygen permeability coefficient across domains created in fiber cell membranes by the high cholesterol content and by densely packed integral membrane proteins indicates that the boundary and trapped lipid domains constitute the major barrier to oxygen permeation [41]. Data show that the oxygen permeability coefficient across the bulk plus boundary domain is smaller by ~30% in nuclear membranes than in cortical membranes [27]. In the case of trapped lipid domains, this difference is ~45% [27]. The oxygen permeability coefficient of the trapped lipid domain in cortical and nuclear membranes is ~4.7 and ~8.5 times smaller, respectively, than the permeability across water layers of the same thickness as the domain. Thus, the trapped lipid domain forms a major membrane barrier for oxygen transport into the lens center; this barrier is significantly greater in the lens nucleus and, as already mentioned, increases with age [27].

It should be mentioned that proteins are nearly impermeable to oxygen [40]; therefore, they are effective barriers to oxygen permeation across the intact fiber cell plasma membrane. As the protein content increases with age [31,32,33], one can conclude that the fiber cell membrane constitutes a barrier to oxygen permeation that grows with the cell age [42]. Finally, it is justified to state that the age-related changes in the lens lipid and protein composition and membrane lateral organization are synchronized so as to increase the resistance of the fiber cell membrane to oxygen permeation, which helps maintain lens transparency and protect it against cataract formation.

## 6. Resistance of Lens Components to Oxidation

The lipid composition of the membranes of lens fiber cells is tightly regulated and, in contrast with other tissues, is independent of diet [43]. There is no turnover of phospholipids, sterols, or proteins in old fiber cell membranes [44,45,46]. Thus, oxidative damage to lipids accumulates with age. Age-related changes in the phospholipid and cholesterol content make fiber cell membranes more resistant to lipid peroxidation and formation of free radicals within the lens. This resistance is greater in the lens nucleus than in the cortex. This is because the sphingolipid content, including dihydrosphingomyelins and sphingomyelins, increases with age at the expense of glycerophospholipid, phosphatidylcholine, and phosphatidylethanolamine [23,26,44,47,48]. In mature fiber cell membranes, ~66 mol% of phospholipids are sphingolipids as compared with ~33 mol% in young cells. Also, saturation of the phospholipid acyl chains increases. Sphingolipids, especially dihydrosphingomyelins, are more saturated than glycerophospholipids, which makes them more resistant to oxidation [24,26,49]. Ravandeh et al. [50] recently published a paper, titled “Protective role of sphingomyelin in eye lens cell membrane model against oxidative stress,” that is perfectly relevant to this section. The three major saturated or monounsaturated fatty acids in mature lens membranes are palmitic, oleic, and nervonic. They account for more than 90% of the total fatty acids [28,49]. Palmitate is the most abundant acyl chain of both sphingolipids (40%) and dihydro-sphingolipids (55%) [24,26]. The decrease in the relative abundance of oleate found in deeper regions of the lens conforms to the observed disappearance of glycerophospholipids in the regions. The concomitant increase in palmitate and nervonate is due to the relative increase in the sphingolipids [28,49].

Human lens fiber cells are considered the longest-living cells in the human body because of their minimal turnover [51]. Also, there is no protein turnover, as proteins cannot be transported from an old lens center to a young cortical area or vice versa [52]. Thus, lens proteins should perform the same functions independently of their age. To maintain lens transparency, effective mechanisms that protect against the accumulation of medicated proteins and those damaged by oxidation with age are needed. Certainly, the regulation of oxygen partial pressure around and inside the lens is one such mechanism.

## 7. Disturbing the Oxygen Partial Pressure around the Lens Promotes Cataract Development

All the indicated mechanisms to control the oxygen partial pressure around and inside the lens help maintain lens transparency through the human life. Any disturbance of these mechanisms causes lens opacification due to the oxidation of fiber cell membrane components [53,54] and cytosolic proteins [55]. The most common disturbance is an acute and/or chronic increase in the oxygen partial pressure around the lens during vitrectomy [20,56] and hyperbaric oxygen treatments [57,58,59]. It was shown that just after vitrectomy, the oxygen partial pressure on the posterior lens surface sharply increases up to 70 mmHg [20,56]. This increase stabilizes months later, as a chronic increase, to ~13 mmHg [20]. This increase is a frequent cause of nuclear cataracts. Also, exposure of the eye lens to high oxygen partial pressure during hyperbaric oxygen therapy leads to nuclear cataract development in most patients [58]. Interestingly, vitrectomies do not cause an increase in the oxygen partial pressure on the anterior surface of the lens.

The existing data indicate that degeneration of the vitreous body with age might contribute to the development of age-related nuclear cataracts. With age, the extent of vitreous liquefaction increases, which is often accompanied by nuclear opacity. Interestingly, neither cortical nor posterior subcapsular cataracts were associated with vitreous body degeneration. Thus, it is concluded that the intact vitreous gel body protects the lens from developing nuclear cataracts. The mechanism of this age-related nuclear cataract development is related to the fact that with the increased liquefaction of the vitreous gel, the concentration of ascorbate is significantly lowered as compared with the intact vitreous gel. As a result, oxygen consumption by the vitreous gel decreases. Animal studies support the mechanism wherein the intact vitreous gel with a high level of ascorbate protects the lens from oxidation [60,61,62].

There are several types of cataracts, including age-related, traumatic, and metabolic. Age-related cataracts are the most common type, but their pathogenesis is multifactorial [63,64,65], and this is outside the scope of our review. Normally the partial pressure of oxygen in the lens is very low, as we mention in our review, which ensures a low level of reactive oxygen species. In the nucleus of young lenses, the formation of the superoxide anion does not lead to protein damage because of the rapid reduction in protein radicals by glutathione and ascorbate. However, with age, the levels of antioxidants decrease [66,67], and it becomes difficult to protect proteins. The only effective barrier against the oxidative reactions observed in older lenses seems to be the maintenance of a low partial pressure of oxygen in the center of the lens.

## 8. Conclusions

Oxygen conditions in the lens and in the retina, which is on the opposite side of the eye, are very different. Thus, the mechanisms that protect the retina are different from those in the lens. Although this is not the subject of this review, the oxygen conditions and the protection mechanisms of both organs are compared below.

1. Oxygen partial pressure (oxygen concentration) within the lens is very low. Oxygen is delivered to the lens through diffusion. The retina is a very well oxygenated system, with oxygen delivered constantly through the blood vessels.
2. Regulation of the oxygen partial pressure is the major mechanism protecting the lens against oxidative stress (as was discussed throughout the review). In the retina, molecular oxygen is involved in the creation of all vulnerable conditions for retinal elements, indicated below, with protective mechanisms developed during evolution to diminish the harmful effects of high oxygen partial pressure in the retina.
3. The lens is avascular with minimal metabolism, and metabolites are delivered to the center of the lens through diffusion. The very high metabolism in the retina requires continuous and intensive delivery of metabolites from the blood. Two major factors protect the lens against the harmful effects of the inflammatory cascade, which can be initiated by cholesterol microcrystals in the cells of other tissue and organs [68,69,70]: First, lens fiber cells lose their intracellular organelles (including inflammasomes) soon after they are formed [1,3], and cholesterol microcrystals cannot activate inflammasomes. Second, the lens is avascular; so, development of an inflammatory cascade is not possible. Thus, cholesterol crystals that are formed in the aged lenses [27] do not disturb lens homeostasis. Although the inflammatory cascade is directly connected with oxidative stress [71], we do not discuss it in this paper. In the retina, the inflammatory cascade can be harmful, as in the case of wet age-related macular degeneration [72,73].
4. Both the lens and the retina are exposed to light. This can create strongly damaging oxidative stress conditions. A healthy lens is transparent and does not contain light-absorbing molecules, especially photosensitizers. Conversely, the function of the photoreceptors in the retina is to absorb incoming light. The retina also contains a number of photosensitizers such as all-*trans* retinal, cytochrome *c* oxidase, and porphyrins [74,75,76,77]. They absorb light and consequently can generate reactive oxygen species and free radicals that can start a damaging oxidative cascade. To decrease the exposure of the retina to the most damaging blue light, macular carotenoids evolved as a blue light filter [78,79]. To increase the effectiveness of this indirect antioxidation action, the pre-receptoral layers of the retina contain a high concentration of macular carotenoids [78,79,80].
5. The lipids of the lens membranes are highly saturated to resist oxidation (see Section 5). In contrast, to maintain the proper functioning of the photoreceptor machinery, photoreceptor membranes are highly unsaturated with high amounts of easily oxidized polyunsaturated phospholipids.
6. To protect the retina, in the membranes of retinal pigment epithelium and photoreceptors, raft domains enriched in saturated lipids and cholesterol are present [81,82,83,84]. Raft domains are surrounded by the bulk domain enriched in long-chain (C18–C24) [85,86,87] and very-long-chain (>C24) polyunsaturated phospholipids with 3–9 double bonds [88,89]. Rhodopsin is also located in the bulk domain of the photoreceptor outer segment membrane [81,82,86,90]. Interestingly, macular carotenoids are essentially excluded from raft domains and concentrate in bulk lipid [91,92]. In this location, they can effectively protect vulnerable polyunsaturated phospholipids and rhodopsin through their antioxidant action, according to the most accepted mechanism through which macular carotenoids, lutein and zeaxanthin, protect the retina from age-related macular degeneration [93,94,95,96].
7. Fiber cells (especially those in the lens nucleus) are considered the longest living cells in the human body (see Section 5). In contrast, the lifespan of photoreceptors is only a few weeks.

As discussed in Section 6, the lipids of fiber cell membranes, as well as the proteins of the cytosolic component, are resistant to oxidation. This, together with the very low oxygen partial pressure in the lens ensure lifelong stability of the fiber cells. The photoreceptors in the retina are highly vulnerable to oxidation. This is because the unsaturated phospholipids, photosensitizers, high concentration of oxygen, and exposure to intensive light focused on the retina by the lens create conditions for the formation of active oxygen species and free radicals. Thus, damage to the photoreceptor is unavoidable. To deal with that problem, evolution shortened the life of the damaged photoreceptor.

In summary, the major mechanisms that were developed during evolution to protect the eye lens against opacification and, thus, against the development of cataract are described in this review. These mechanisms are based on the regulation of the oxygen partial pressure both outside and inside the lens; so, the effective oxygen concentration within the lens is very low. The mechanisms are unique to the lens because, to the best of our knowledge, they are not used by other tissues or organs of the human body. In a normal lens, these mechanisms work throughout the entire human life; any disturbance to these mechanisms results in the development of cataract.

## Data Availability

Not applicable.
